# Fatal trauma: death anlysis from September 2011 to December 2013, at Fortis hospital, Noida, India

**DOI:** 10.1186/1865-1380-8-S1-P1

**Published:** 2015-04-22

**Authors:** Rinkey Ahuja, Keshav Singh, Rizwan Gani, Dina Shah;

**Affiliations:** 1 Fortis Hospital, Noida, India

## Objectives

Fortis hospital, Noida is the tertiary care referral centre for trauma in Western Uttar Pradesh. The hospital implemented trauma teams in 2011. The purpose of this study is to look at the modal distribution of deaths in cases of major trauma to improve trauma care at our institution and to provide comparative data for other institutions.

## Methods

A retrospective 2-year review from September 2011 to December 2013 was conducted using a database from medical records of the emergency department. Review of patient records included: population demographics, mode of injury, delay in arrival if any, length of stay prior to death, and the cause of death.

## Results

4226 trauma patients visited emergency department. 2208 (52.24%) patients were discharged, 1537 (36.37%) were admitted to the ICU, and 454 (10.74%) were admitted to the ward. The remainder were Left Against Medical Advice (LAMA) or Dead on Arrival (DOA) due to various reasons, and there were 27(0.63%) deaths in emergency department (within 15 min of arrival) due to haemorrhagic and/or neurogenic shock.

Of the patients who were admitted in ICU or ward, 48 (1.1%) patients died in the hospital.

As per our analysis of those 1.1% patients who died in hospital;

• Majority of patients (71%) were in the age group of 20 to 40years. Male to female ratio was 87:13%.

• 41 patients (85.4%) came after more than one hour of injury while only 7 patients (14.5%) came within one hour.

• The most common causes of injuries in those patients were road traffic accidents (77%), compared to gunshot wounds (8.3%), fall from height (12.5%) and blunt injuries (2.1%).

• 9 (18.75%) patients died within 24 hours of the hospital stay, 19 (39.5%) died within 72 hours, and 18 (33.3%) died more than 72 hours after admission.

• Most frequent cause of death in our observational study was severe head injury in 22 patients (45.8%), multiple organ failure/sepsis in 10 patients (20.83%), and exsanguination in 6 patients (12.5 %). 10 patients (20.83%) died because of combination of causes.

## Conclusions

• Road traffic accidents and their delayed presentations caused a majority of trauma deaths in our observational study of two years. This highlights the need for developing improved emergency care at local healthcare facilities, development of trauma centers, and adoption of transfer protocols for these trauma patients centres to reduce morbidity and mortality.

